# Using clinical trial data and linked administrative health data to reduce the risk of adverse events associated with the uptake of newly released drugs by older Australians: a model process

**DOI:** 10.1186/1471-2458-11-361

**Published:** 2011-05-21

**Authors:** Margaret T Whitstock, Christopher M Pearce, Stephen C Ridout, Elizabeth J Eckermann

**Affiliations:** 1 Department of Sociology, School of History, Heritage and Society, Deakin University, Pigdons Road, Geelong, Vic 3217, Australia; 2 Faculty of Medicine, Nursing and Health Sciences, Monash University, Wellington Road, Clayton, Vic 3800, Australia; Melbourne East General Practice Network, 250 Mont Albert Road, Surrey Hills, Vic 3127, Australia; 3 School of Population Health, University of Western Australia, 35 Stirling Highway, Crawley, Perth, WA 6009 Australia

## Abstract

**Background:**

The study was undertaken to evaluate the contribution of a process which uses clinical trial data plus linked de-identified administrative health data to forecast potential risk of adverse events associated with the use of newly released drugs by older Australian patients.

**Methods:**

The study uses publicly available data from the clinical trials of a newly released drug to ascertain which patient age groups, gender, comorbidities and co-medications were excluded in the trials. It then uses linked de-identified hospital morbidity and medications dispensing data to investigate the comorbidities and co-medications of patients who suffer from the target morbidity of the new drug and who are the likely target population for the drug. The clinical trial information and the linked morbidity and medication data are compared to assess which patient groups could potentially be at risk of an adverse event associated with use of the new drug.

**Results:**

Applying the model in a retrospective real-world scenario identified that the majority of the sample group of Australian patients aged 65 years and over with the target morbidity of the newly released COX-2-selective NSAID rofecoxib also suffered from a major morbidity excluded in the trials of that drug, indicating a substantial potential risk of adverse events amongst those patients. This risk was borne out in post-release morbidity and mortality associated with use of that drug.

**Conclusions:**

Clinical trial data and linked administrative health data can together support a prospective assessment of patient groups who could be at risk of an adverse event if they are prescribed a newly released drug in the context of their age, gender, comorbidities and/or co-medications. Communication of this independent risk information to prescribers has the potential to reduce adverse events in the period after the release of the new drug, which is when the risk is greatest.

Note: The terms 'adverse drug reaction' and 'adverse drug event' have come to be used interchangeably in the current literature. For consistency, the authors have chosen to use the wider term 'adverse drug event' (ADE).

## 1. Background

Coping with the outcomes of adverse drug events (ADEs) associated with the use of newly approved drugs, particularly in the elderly, has become a significant problem in both primary care and hospital settings in Australia, and is a major challenge for public health policy worldwide. One in every ten Australian patients who attend a GP in a primary care consultation has experienced an ADE in the preceding six months, and one in twenty has experienced a moderate to severe ADE [1]. This translates to an estimated number of 2 million Australians who experience an ADE annually, with 1 million of these ADEs classed as moderate or severe, and 138,000 requiring hospitalisation [2]. ADEs occur particularly in the elderly: adverse drug events in people aged 60 years and over which had caused admission to, or an extended stay in, Western Australian hospitals between 1981-2002 increased more than five-fold during that period [3].

Older patients are particularly vulnerable to adverse drug events because of multiple-drug regimens and age-associated changes in pharmacokinetics and pharmacodynamics [4]. Privileging data from randomised controlled clinical trials (RCTs) as the source of best evidence to guide clinical decision-making may not well serve the needs of the increasing number of patients in older age groups, and may add to their problems rather than ameliorating them.

Many older patients suffer from illnesses targeted by newly approved drugs. However factors such as decreasing physiological resilience, the follow-on effects of cumulative morbidities, and effects of the increasing numbers of medications taken to manage these morbidities, together mean that the health profile of older populations is moving further and further away from the characteristics of the populations used in the clinical trials. Despite this lack of fit, it is patients in older age groups who are most frequently being prescribed new drugs as soon as they are approved. The potential for adverse drug events is greater when a new drug is prescribed soon after its release onto the market, and rapid uptake after release can place patients at risk of adverse drug reactions and serious drug interactions through co-prescribing [5].

A recent Australian medical journal editorial called for the development of computerised systems that can deal with the reality that only limited numbers of highly selected patients are studied before a drug is approved for marketing [2]. One such system could be an improved pharmacovigilance system to detect ADE patterns associated with a newly prescribed drug; however, identifying the offending drug is not easy in a multi-morbidity/multi-drug context, and pharmacovigilance systems also have an inbuilt epistemological problem, as the reporting process is subject to Type I and Type II errors [6].

Pharmacovigilance systems rely on spontaneous reporting, and the large majority of adverse events are either not recognised or not reported [7,8]. It has been estimated by the FDA that the MedWatch system receives only between 1% to 10% of adverse drug event reports associated with any given drug [7-9]. Spontaneous reporting of adverse events has been described as a particularly poor instrument for detecting increased risks of common conditions such as cardiac disease [10,11]. These common conditions are often those diseases which become chronic in older age groups, and it is persons in these age groups who are increasingly suffering the effects of the impact of new drugs in the context of old morbidities. A post-hoc approach to identifying drug risks is too late for many older persons.

The Australian medical journal editorial referred to above identified the need for a computerised system that could focus on detecting early signals of potential ADEs [2]. This paper presents a three-part risk identification process that can be used to forecast which patient groups could potentially be at risk of an ADE if they are prescribed a newly released drug. If this process is undertaken before any newly released drug is approved for inclusion as a subsidised drug via Australia's Pharmaceutical Benefits Scheme (PBS), this will provide an opportunity to significantly reduce the numbers of persons who will need treatment for new-drug-associated ADEs. Shifting costs away from responding to preventable adverse health events and towards preventing these adverse health events from occurring in the first place is the preferable option for public health expenditure.

## 2. Methods (Part 1)

This section addresses the generic methods underlying the proposed risk identification model. The section entitled Methods (Part 2) illustrates the application of these methods in the case of the clinical trials of the COX-2-selective NSAID rofecoxib and the patient groups who would have been the main target population for that arthritis drug.

### 2.1 Risk identification using clinical trial data

The first step uses data from reports of RCTs of a new drug. The RCT study design relies on the exercise of a range of controls to minimise bias, and this is done in order to achieve the internal validity that is seen as a scientific gold standard. However, internal validity is often achieved at the expense of external validity. The important issue of concern for older Australians taking new drugs is that though more within-trial controls contribute to an internally valid result, these controls frequently involve the exclusion of potential participants who have the very comorbidities and co-medications that commonly exist in older age groups.

The RCT reports of a new drug are examined to identify whether there are age groups, gender, comorbidities and/or co-medications recorded as having been excluded from these clinical trials. Once exclusions are applied in an RCT, for whatever reason, this removes the possibility of gathering evidence on how the new drug will act in patients who, for example, suffer from one or more of the excluded morbidities, and/or who take one or more of the excluded medications.

The examination of reports of clinical trials of new drugs should take into consideration the scope of the research questions posed, what data were sought, from whom they were sought, what endpoints were adjudicated, and what were the bases for outcome analyses. In this process, it is important to differentiate between those exclusions which are specifically related to the research questions being asked, and those exclusions which relate to other body systems. For example, a clinical trial of a new drug which is being evaluated for its specific effects on gastric mucosa may have a protocol that excludes persons already suffering from gastric morbidities such as ulceration or bleeding. The exclusion of patients with such gastric morbidities relates specifically to the research question which seeks to evaluate the new drug in relation to its effects on gastric mucosa. However, exclusion protocols relating to morbidities in other body systems are applied for different reasons, and can reduce the availability of important knowledge about the action of the new drug in patients who have those excluded morbidities.

Access to information on the clinical trials of a new drug is via published research papers, but these may not always be available, as sometimes they can be withheld by the drug developer until after the drug is approved for marketing. The Australian Therapeutics Goods Administration (TGA) considers the results of Phase 1, 2 and 3 trials for a drug before it approves the registration of that drug for marketing in Australia. TGA approval is also a pre-requisite for any drug to be considered for inclusion in the PBS schedules. The TGA has recently commenced publishing an Australian Public Assessment Report (AusPAR) on each drug that is approved for marketing in Australia. These AusPAR reports are a valuable source of information as they include detailed summaries of the steps in the evaluation process that led the TGA to approve or not approve a prescription medicine submission. AusPARs are modelled on the similar EPAR public assessment report system implemented in the European Union [12].

Another significant source of RCT data is the U.S. National Institutes of Health (NIH) website. Since 2007, U.S. legislation has mandated that information about all clinical trials that are commenced in the U.S. must be made available on the NIH website with 2 months of the enrolment of the first patient. The sponsor or developer of the new drug must submit to the NIH data which include, *inter alia*, descriptive information, in language intended for the lay public, concerning the purpose of the trial, the study design, the disease or condition being studied, the name of the drug, primary and secondary outcome measures, eligibility criteria, including exclusion protocols, and the trial protocol identifier. The results of all clinical trials carried out in the U.S. are required by law to be made available on the NIH website within 30 days of the approval of a new drug by the U.S. Food and Drug Administration (FDA). These results must include data on participating patient demographics, withdrawals and exclusions from the final analysis, and tables of values for primary and secondary outcome measures, including statistical analyses [13]. The NIH clinical trials database also includes reports of clinical trials sponsored in other jurisdictions when those trials have U.S. trial arms [14]. Some 50% of the world's new drugs are launched in the U.S. [15,16], so the NIH website will be a comprehensive source of detailed information on clinical trials of new drugs. At the time of writing, the NIH website contains information on 88,402 trials with locations in 172 countries.

The outcome of the first part of the model process is a risk profile of patient groups whose ages, gender, comorbidities and co-medications were not included in the trial, and about whom there are effectively no data on the potential action and potential effects of that new drug.

### 2.2 Risk identification using Australian patient data

The second step uses de-identified administrative health data to establish the age groups and gender of selected subsets of current real-world Australian patients (e.g. patients over 65 years of age) who have the morbidity targeted by the new drug, and to establish what are the comorbidities suffered by these patients and what are the co-medications they are being prescribed. The process uses morbidity data drawn from the diagnoses fields of hospital separations records which have been linked with medications data drawn from PBS pharmaceutical dispensing records.

Under a Memorandum of Understanding between the Western Australian Department of Health and the Commonwealth Department of Health and Ageing, the W.A. Department of Health Data Linkage Unit has established linkages between that State's hospital separations records and the PBS dispensing records held by the Commonwealth Department of Health and Ageing. Using the linked data, it is possible to establish which patient groups have the morbidity targeted by a new drug (either via a hospital diagnosis and/or via one or more medications dispensed), and then establish what are the co-morbidities and co-medications associated with these patients (again, either via hospital diagnosis and/or medications dispensed). The result is a multi-source health profile of patients with the morbidity of interest.

Linked morbidity data can also be sourced from the Commonwealth Department of Health and Ageing Medicare rebate data for approved consultations, procedures and diagnostic services. Using these data would improve and broaden the health profiling process. However, we were looking for proof of the concept, and worked only with two datasets described above.

Part of the remit of the newly formed Australian Population Health Research Network is to link de-identified information from key administrative health datasets across State, Territorial and Commonwealth jurisdictions and health sectors to support population-based research. The anticipated outcome benefits are the capacity to improve the safety and quality of health care, assess the effectiveness of preventive interventions, enable longitudinal follow-up of research studies and surveys, and monitor trends in the patterns and costs of health care [17,18]. The authors suggest that development of Australia-wide risk profiles for patient groups with the target morbidities of newly released drugs could become one of the responsibilities of this new body.

### 2.3 Risk identification comparing clinical trial data and patient data

The third step is to take the comorbidity and co-medications risk profile developed from the examination of exclusion protocols applied in RCTs of a new drug, and compare it with the comorbidity and co-medications risk profile developed from examination of the selected subsets of current real-world Australian patients who have the morbidity targeted by the new drug. Where these two risk profiles differ, for example, when there is one or more factors excluded in the clinical trials but present in the real-world population, there is likely to be limited information available about the action of the new drug in those real-world patient groups. The output of the comparison is a risk profile for the selected subsets of current Australian patients who have the morbidity targeted by a new drug, but who could potentially be at risk of an adverse drug event if they are prescribed that new drug in the context of their current age, gender, comorbidities and/or co-medications. The final output of the process is a warning advisory to be circulated to prescribers warning of the lack of information on the action of the new drug in the selected subsets of the national population, and warning that if the drug is prescribed, to closely monitor those patients to whom it is prescribed.

When a new drug targets a morbidity experienced by a large number of older patients, implementing the model to assess potential risk should be a priority if the drug is proposed for listing in the PBS schedules, as this is usually the trigger for wide uptake.

## 3. Methods (Part 2)

This section presents a practical application of the generic methods outlined in Methods (Part 1). The data analysed are from 14 clinical trials of the COX-2-selective NSAID rofecoxib, and the comorbidities and co-medications of an arthritis patient cohort aged 65 years and over in the State jurisdiction of Western Australia. These patients formed part of the potential receiving population for rofecoxib.

### 3.1 Risk identification using data from clinical trials of rofecoxib

We analysed clinical trial data from 14 RCTs of the COX-2-selective NSAID rofecoxib which were indicated as being available in Australia before February 2001, when rofecoxib was included in the PBS schedules [19-22], which was the trigger for rapid uptake of the drug across Australia. The 14 studies include eleven efficacy trials, two safety trials, and one pooled study drawing data from eight of the eleven efficacy trials [23-36].

The clinical trials of rofecoxib were checked for information on the trial category (safety/efficacy), trial design, aims and endpoints adjudicated, number of participants, their age range and health status, excluded comorbidities and co-medications, rofecoxib and comparator dosage levels, trial durations, and patient withdrawals. The principal focus was on the comorbidities and co-medications which disqualified arthritis patients from participation in the nine efficacy trials for patients with osteoarthritis (OA) and the two efficacy trials for patients with rheumatoid arthritis (RA).

Persons of both genders aged 65 years and over, the age group of interest to us, were represented in the RCTs analysed. We took into account that the COX-2-selective NSAID rofecoxib was developed specifically to relieve the pain and inflammation of arthritis without the gastrointestinal morbidity associated with the use of non-selective NSAIDS to treat arthritis inflammation. As expected, there were consistent applications of gastric morbidity exclusion protocols specifically related to enabling the research questions to be asked. There were also applications of exclusion protocols related to enabling assessment of whether use of rofecoxib resulted in improvement in arthritis symptoms. For example, in an RCT evaluating rofecoxib for its specific effects on improving pain on walking, exclusion protocols required the capacity to walk for some measurable distance in order to be able to evaluate improvement.

Of the exclusions relating to other body systems, we also noted the exclusion of participants who had suffered an unspecified active or recent hepatic disease. We recognised that this exclusion was related to ensuring the bioavailability of the new drug to be trialled. In retrospect, it could have been of value to extract de-identified patient data on the level of occurrence of hepatic disease in the over-65 age group with OA or RA. We also noted the consistent exclusion of prospective participants who had suffered one or more of a range of neoplastic events.

However, of exclusion protocols relating to other body systems, what stood out in the examination of the reports on the clinical trials of rofecoxib was the exclusion of participants with vascular morbidities, and the exclusion of participants who were taking vascular medications. Patients with OA were excluded from participation in the analysed RCTs of rofecoxib if one or more of the following morbidities applied: adverse cardiovascular factors (including Class III/IV angina or congestive heart failure, controlled or uncontrolled hypertension, or a requirement for anti-coagulant therapy), adverse cerebrovascular factors (including a stroke within 2 years prior, or a transient ischaemic event within 2 years prior), or severe renal impairment (creatinine clearance ≤ 30 ml/min). Patients with RA were excluded from participation in the analysed RCTs of rofecoxib if one or more of the following morbidities applied: adverse cardiovascular factors (including a requirement for anti-coagulant therapy, or a myocardial infarction within 1 year prior), adverse cerebrovascular factors (including a stroke within 2 years prior, or a transient ischaemic attack within 2 years prior), or severe renal impairment (creatinine clearance ≤ 30 ml/min).

**Figure 1:**
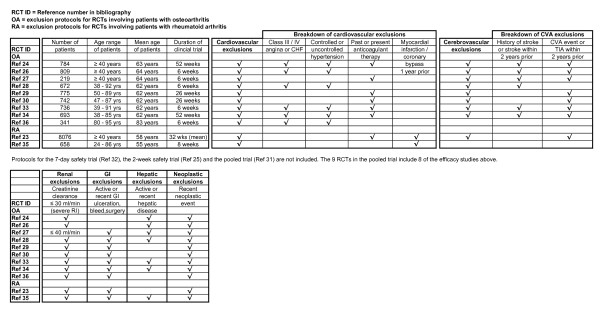
**Main comorbidity exclusion protocols applied in the efficacy clinical trials of rofecoxib, by arthritis type**.

**Figure 2:**
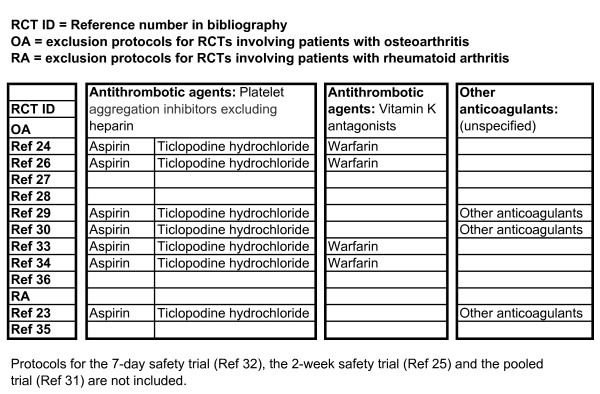
**Vascular co-medication exclusion protocols applied in the efficacy clinical trials of rofecoxib, by arthritis type**.

Figure 1 presents the main comorbidity exclusion protocols applied in the efficacy clinical trials of rofecoxib, grouped by arthritis type, and Figure 2 presents the vascular co-medications exclusion protocols applied in the efficacy clinical trials of rofecoxib, also grouped by arthritis type.

This exclusion of patients being treated for cardiovascular (CVD), cerebrovascular (CVA) or renal disease meant that, as at February 2001, when rofecoxib was made available through the PBS, there were effectively no data available on the action of rofecoxib in patient groups who were being treated for these morbidities. It was this lack of data on the action of rofecoxib in the presence of CVD, CVA and renal disease, as applied to those arthritis patients in the 65 years and over age group, that was of interest to the researchers.

### 3.2 Risk identification in an Australian arthritis patient cohort

The real-world current patient groups we selected were W.A. patients, male and female, who were aged 65 years and over in 1999 and 2000, and who had either OA or RA, the two major morbidities targeted by rofecoxib. The presence of either OA or RA was to be established based on de-identified PBS medications and hospital morbidity data, and co-morbidities and co-medications data were to be established through links to the OA or RA morbidity or medications data. The years 1999 and 2000 were chosen as arthritis patients in these two year cohorts would have formed the potential target population for rofecoxib.

The project was granted institutional ethics approval by Deakin University, and this was accepted by the W.A. Department of Health (W.A. DoH) and the Commonwealth Department of Health and Ageing (DoHA).

### 3.2.1 Medications data requested

Using the Australian Statistics on Medicine, we selected a list of PBS Item Codes for high-use OA and RA medications dispensed in 1999 and 2000, and submitted the list to DoHA through the W.A. DoH Data Linkage Unit (DLU). We requested all PBS Item Codes linked to the patients, male or female, aged 65 years and over, who had been hospitalised with arthritis, as identified by a selected list of ICD-9-CM and ICD-10-AM arthritis diagnosis codes (see 3.2.2 below). We also requested person-level PBS dispensing data for non-hospitalised patients dispensed any of the selected high-use OA or RA medications, together with any other co-medications dispensed to these patients. We further requested data on all medications that had been dispensed in the previous year to those patients in the Year 1999 and Year 2000 cohorts who had been prescribed the selected OA or RA medications. Data supplied included age and gender variables.

We received 1,370,073 PBS dispensing records definitively linked to W.A. patients aged 65 years and over who had been prescribed one or more of the selected OA or RA medications in 1999, together with data on any medications dispensed to these patients in 1998, plus any PBS medications that had been dispensed to W.A. patients aged 65 years and over who had an OA or RA hospital diagnosis in 1999. We also received 1,817,455 PBS dispensing records definitively linked to W.A. persons aged 65 years and over who had been prescribed one or more of the selected OA or RA medications in the year 2000, together with data on any medications dispensed to these patients in 1999, plus any PBS medications dispensed to W.A. patients aged 65 years and over who had an OA or RA hospital diagnosis in 2000.

We also requested de-identified data for all the PBS medications dispensed to any patients who were prescribed rofecoxib between January 2003 and the end of September 2004, when rofecoxib was withdrawn for safety reasons, as we wished to examine what medications were being co-prescribed to patients who were prescribed rofecoxib during this period. We received 3,561,701 PBS medications records for this analysis.

Though the medications data for the 1999 cohort were analysed, this paper will report only on the detailed analyses of the medications data from the 2000 cohort, the year prior to the inclusion of rofecoxib in the PBS schedules. As noted in the key outcomes discussed in Section 5, there was a difference of only 1.5% between the year 1999 and the year 2000 medications analyses. The decision to restrict the 1999 medications reporting was linked to issues associated with a change in ICD coding in that year in Western Australia (see 3.2.2 below), as the final cohort analyses for each year were designed to integrate both medications and morbidity data.

Analysis of the January 2003 - September 2004 medications data is also addressed in Section 5.

### 3.2.2 Morbidity data requested

Using International Classification of Diseases (ICD) manuals, we selected a list of ICD-9-CM arthritis codes (for Jan-July 1999) and ICD-10-AM arthritis codes (July 1999 - Dec. 2000) which was submitted to the W.A. DoH DLU. The extraction specifications requested person-level hospitalisation morbidity data for all Year 1999 and Year 2000 patients, male or female, aged 65 years or over whose hospital separation records included any of the selected OA or RA ICD codes in either the primary diagnosis field or in any secondary diagnosis field. We also requested hospital morbidity data from any previous year's hospitalisation for those 1999 and 2000 patients.

For the 1999 cohort, we received 27,580 hospital morbidity records definitively linked to W.A. patients aged 65 years and over. These records included hospital morbidity data from any patient hospitalisation episode in 1999, the previous year's hospital morbidity data for any 1999 patient who had a hospitalisation episode in 1998, plus 1999 hospital morbidity data linked to any patients who had been dispensed a selected OA or RA medication in 1999. For the 2000 cohort, we received 34,269 hospital morbidity records definitively linked to W.A. patients aged 65 years and over. These records included hospital morbidity data from any patient hospitalisation episode in 2000, the previous year's hospital morbidity data for any 2000 patient who had a hospitalisation episode in 1999, plus 2000 hospital morbidity data linked to any patients who had been dispensed a selected OA or RA medication in 2000.

On analysis, the 1999 data showed significant inconsistencies at the rollover from ICD-9-CM to ICD-10-AM. The difference appears to be associated with a greater frequency of application of secondary arthritis diagnoses under ICD-9-CM, or a lower frequency of application of secondary arthritis diagnoses under ICD-10-AM. Given this inconsistency, we continued analysis with morbidity data from the year 2000 only.

## 4. Results

This section describes the analysis of the medication and morbidity data to support the identification of patients aged 65 years and over who would have potentially been at risk if they had been prescribed rofecoxib. It includes analyses of prescription medications dispensed to this age cohort in 2000, and hospital-confirmed morbidities experienced by this age cohort in 2000.

### 4.1 Medications data analysis - 2000 cohort

The 1,817,455 dispensing records for the Year 2000 cohort were sorted into two groups: those for selected OA and RA medications, and all other medications. Using the Australian Statistics on Medicine, we selected a list of high-use CVD medications dispensed in 2000, and using this list we sorted all the non-OA/RA medications into three sub-groups: selected CVD medications, all medications dispensed ≥3,000 times, and medications dispensed <3,000 times. All medications dispensed <3,000 times were not further considered.

There were 235,088 prescriptions dispensed for the selected OA and RA medications, 514,756 prescriptions dispensed for the selected CVD medications, and 653,793 prescriptions in the category of medications dispensed ≥3,000 times.

Further analysis focused on the CVD medications group, as CVD medications and morbidities were subject to exclusion protocols in the RCTs of rofecoxib which were available prior to February 2001, when rofecoxib was included in the PBS schedules. All OA/RA medications and CVD medications were analysed to develop a person-level summary of these medications as dispensed in 2000.

Of the 58,968 patients in the 2000 cohort aged 65 years and over who were prescribed OA/RA medications, 40,495 were co-prescribed CVD medications (see Table 1). In the 2000 cohort, 68.8% of patients were being medicated for both OA/RA and CVD.

**Table 1:** 2000 Cohort/Patient Medications

Drugs Prescribed	No. of patients	% of total
OA/RA drugs only	18,373	31%
OA/RA plus CVD drugs	40,595	69%
Total	58,968	100%

Table 2 presents and age and gender breakdown of the 18,373 patients taking OA/RA medications only and the 40,595 patients who were co-prescribed OA/RA medications plus CVD medications. The high percentage (61%) of the 65-69 year age group co-prescribed arthritis and cardiovascular medications would indicate that the co-prescribing was already well established in this Australian cohort by the age of 65, and that cardiovascular disease was a significant morbidity for many arthritis patients as they aged. The co-prescribing of OA/RA and CVD medications compared with only OA/RA medications increased steadily in all age groups.

**Table 2:** 2000 Cohort / OA/RA vs. OA/RA + CVD medications, by age and sex

Age Group	OA/RA drugs only Male	OA/RA drugs only Female	% of patients taking OA/RA drugs only	OA/RA plus CVD drugs Male	OA/RA plus CVD drugs Female	% of patients taking OA/RA plus CVD drugs
65-69	2,862	3,272	39%	4,427	5,255	61%
70-74	2,528	2,607	32%	4,999	6,035	68%
75-79	1,707	1,980	28%	4,091	5,535	72%
80-84	854	1,175	26%	2,159	3,648	74%
85-99	491	897	24%	1,327	3,119	76%
Sub-totals	8,442	9,931		17,003	23,592	

	Patient Total = 18,373			Patient Total = 40,595		

These statistics suggest that the prescribing of a new arthritis drug which consistently excluded persons with cardiovascular disease from participating in its clinical trials carries an elevation of risk if patients are moving into the older age groups where it is evident that cardiovascular risk is increasing. The declining ratio of males to females in the older age groups co-prescribed OA/RA and CVD drugs suggest an additional elevation in cardiovascular risk for males.

### 4.2 Morbidity data analysis - 2000 Cohort

The 34,269 Year 2000 hospital morbidity records included data for 5,321 patients with an OA or RA diagnosis, and 28,948 patients with diagnoses for other morbidities (see Table 3).

**Table 3:** 2000 Cohort/Hospital morbidities by morbidity type

Morbidity	No. of patients	% of total
OA/RA morbidities	5,321	16%
Non-OA/RA morbidities	28,948	84%
Total	34,269	100%

The age by sex breakdown of the 5,321 patients with an OA or RA diagnosis is given in Table 4. The distribution of male and female patients with hospital diagnoses of OA/RA morbidity diverge from near-parity in the 65-69 year age group to a 1:3 survival rate in the 85-99 age group. These statistics suggest that the prescribing of a new arthritis drug which had consistently excluded persons with cardiovascular disease from participating in its clinical trials carries more risk for Australian male patients moving into the older age groups.

**Table 4:** 2000 Cohort/Patients with OA and RA morbidities, by age, by sex

Age Group	Total numbers with OA/RA	Sex = Male	Males as % of age group	Sex = Female	Females as % of age group
65-69	1,142	553	48%	589	52%
70-74	1,259	557	44%	702	56%
75-79	1,230	465	38%	765	62%
80-84	836	294	35%	542	65%
85-99	854	217	25%	637	75%

	Total = 5,321	Total = 2,086		Total = 3,235	

The 34,269 hospital morbidity records were then merged with the 58,968 medications records patient OA/RA medications records for the 2000 cohort merged to support analyses of the common links. 44% of the Australian patients over 65 years who suffered from OA/RA diseases were treated with OA/RA medications only; 4% of patients showed as having only a hospital diagnosis of OA/RA, with no OA/RA medications (and we surmise that these patients may be taking other analgesics such as paracetamol or acetaminophen); 5% of patients who were prescribed OA/RA medications and also had an OA/RA hospital diagnosis; and 47% of patients who were taking OA/RA medications suffered had hospital diagnoses for one or more of cardiovascular, cerebrovascular and renal disease (see Table 5 below).

**Table 5:** 2000 Cohort/Merged OA/RA medications and hospital morbidity data

Total number of patients	61,139	% of total
Patients with OA/RA medications only	26,870	44%
Patients with OA/RA morbidities only	2,171	4%
Patients with OA/RA medications linked to OA/RA morbidities	3,150	5%
Patients with OA/RA medications linked to non-OA/RA morbidities	28,948	47%

		100%

Table 6 reports on those patients in the 2000 Cohort who were prescribed OA or RA medications and who also had a hospitalisation event associated with a non-OA/RA morbidity. Of the 58,968 patients taking an OA/RA medication, there were 3,522 patients who had a hospital CVD diagnosis; 666 patients taking an OA/RA medication who also had a hospital CVA diagnosis; and 551 patients taking an OA/RA medication who also had a hospital diagnosis of renal disease.

**Table 6:** 2000 Cohort/Patients on OA/RA medications who also had CVD, CVA or renal morbidity, by age and sex

	OA/RA drugs plus cardiovascular disease		*Male* *vs*. *Female*	OA/RA drugs plus cerebrovascular disease		*Male* *vs*. *Female*	OA/RA drugs plus renal disease		*Male* *vs*. *Female*
**Age Group**	**Male**	**Female**	**%**	**Male**	**Female**	**%**	**Male**	**Female**	**%**

65-69	356	203	*64 : 36*	49	32	*60 : 40*	32	27	*54 : 46*
70-74	488	303	*62 : 38*	77	47	*62 : 38*	62	29	*68 : 32*
75-79	445	379	*54 : 46*	84	83	*50 : 50*	76	45	*63 : 37*
80-84	327	327	*50 : 50*	78	85	*48 : 52*	66	54	*55 : 45*
85-99	244	450	*35 : 65*	44	87	*34 : 66*	71	89	*44 : 56*

Sub-totals	1,860	1,662		332	334		307	244	

	Patients = 3,522			Patients = 666			Patients = 551		

A comparison of the number of Australian females and males over 65 years who were taking OA/RA medications in the Year 2000 shows that males had significantly higher levels of cardiovascular morbidity in the 65-69, 70-74 and 75-79 age groups, after which the data reflect their relative reduced longevity compared with females. This male/female pattern was repeated with cerebrovascular disease and renal disease. However, the major comorbidity for both sexes is clearly cardiovascular disease. The current morbidity patterns of the population from which local patients were drawn would suggest that the prescribing of a new arthritis drug which had consistently excluded persons with cardiovascular disease from participating in its clinical trials carries an elevated risk for Australian patients over 65 with OA/RA, with this risk being higher for male patients.

Table 7 reports on those patients in the 2000 Cohort who were being treated in hospital for OA or RA who were also treated in hospital for a non-OA/RA morbidity. There were 966 patients treated in hospital for OA or RA who were also being treated for CVD; 227 patients treated in hospital for OA or RA who were also being treated for cerebrovascular disease; and 263 patients treated in hospital for OA or RA who were also being treated for renal disease.

**Table 7:** 2000 Cohort / OA/RA morbidities with CVD or CVA or Renal morbidities

	OA/RA hospital diagnosis + cardiovascular hospital diagnosis		*Male* *vs*. *Female*	OA/RA hospital diagnosis + cerebrovascular hospital diagnosis		*Male* *vs*. *Female*	OA/RA hospital diagnosis + hospital diagnosis of renal disease		*Male* *vs*. *Female*
**Age Group**	**Male**	**Female**	**%**	**Male**	**Female**	**%**	**Male**	**Female**	**%**

65-69	42	34	*55 : 45*	6	6	*50 : 50*	6	14	*30 : 70*
70-74	78	70	*53 : 47*	20	11	*65 : 35*	18	10	*64 : 36*
75-79	79	123	*39 : 61*	22	36	*38 : 62*	30	27	*53 : 47*
80-84	77	154	*33 : 67*	29	37	*44 : 56*	33	40	*45 : 55*
85-99	91	218	*29: 71*	11	49	*18 : 82*	28	57	*33 : 67*

Sub-totals	367	599		88	139		115	148	

	Patients = 966			Patients = 227			Patients = 263		

There were also 163 patients in the 2000 cohort who were treated in hospital for OA or RA who were also being treated for both CVD and CVA; 87 patients being treated for OA or RA who were also being treated for CVD and renal disease; and 13 patients being treated for OA or RA who were also being treated for CVD, CVA, and renal disease.

## 5. Discussion

From the reports of the efficacy RCTs of rofecoxib, it is clear that the study design of these clinical trials significantly curtailed the opportunities for patients with cardiovascular, cerebrovascular and renal comorbidities to be exposed to rofecoxib, and hence restricted the data on the action of rofecoxib in these vascular morbidity contexts.

From the linked de-identified administrative health data for W.A. arthritis patients aged 65 years and over in 2000, it is clear that CVD was a major comorbidity in those patients, particularly in males, as identified in both PBS medications data and hospital morbidity data. In 2000, 68.8% of patients prescribed OA/RA medications were also prescribed CVD medications. The high number of patients in the 2000 cohort who were diagnosed with CVD in hospital underscores this exposure.

Though the detailed analyses of the 1999 medications data are not included in the paper, in that year, 67.3% of patients prescribed OA/RA medications were also prescribed CVD medications.

A pre-2001 analysis of available published clinical trials of the OA/RA drug rofecoxib would have highlighted the limited participation of arthritis patients with cardiovascular morbidity; and an examination of the medications prescribed to real-world Australian OA/RA patients aged over 65 years would have shown that some 68% of these arthritis patients carried a cardiovascular risk. Knowledge of these two factors alone could have provided a clear caution concerning prescription of rofecoxib for this arthritis patient group until more was known about its effects in the context of CVD.

Other vascular morbidities, both cerebrovascular and renal, were also evident in the patient population who formed the potential receiving population for rofecoxib.

In hindsight, we know that the major problem with rofecoxib was its capacity to precipitate or aggravate CVD and CVA morbidity and mortality. Though the risk of cardiovascular events was recognised by the FDA as early as February 2001, this risk was not generally known to prescribers due to the lack of adequate information provided by the manufacturer.

To assess whether this could have been the case, we checked all the medications data for persons dispensed rofecoxib between January 2003 and September 2004 (when rofecoxib was withdrawn) to ascertain what other medications were being taken by those persons during that period. Of the 38,010 persons prescribed rofecoxib in that period, 67% were also being co-prescribed medications to treat CVD, a potential indicator of a lack of awareness of the link between cardiovascular risk and use of the rofecoxib arthritis drug. Prior to its voluntary safety withdrawal in September 2004, it has been estimated that rofecoxib was implicated in more than a thousand ADEs in Australian patients, of which about 30% resulted in deaths [37].

It is essential that there is a timely source of risk information concerning new drugs, and this source must be independent of the product information provided by the new drug's manufacturer, or by representatives of that manufacturer who visit medical practices. Identification of likely or possible risk should be undertaken before there is the opportunity for a problem to be realised.

Had we assembled all the relevant information we now know to have been available in Australia before the PBS listing of rofecoxib, we could have identified the discrepancies between the profile of the OA/RA patients included in the analysed rofecoxib RCTs and the profile of the local OA/RA patient groups aged 65 years and over. The restriction of cardiovascular, cerebrovascular and renal disease exposure in rofecoxib trial patients, and the limitation of cardiovascular adjudication in most of the analysed RCTs, could together have served to indicate a significant shortfall in knowledge about the action and effects of rofecoxib in those disease contexts, and pointed to the potential risk of ADEs in patients with these diseases if they were to be prescribed the drug. These patients could then have been advised to take a different NSAID, paracetamol, or other treatment option. The rofecoxib-associated morbidity and mortality adverse events in Australian patients could have been reduced using data that was available before they occurred.

## 6. Conclusion

Implementing the model process described in this paper before a newly released drug is included in Australian PBS schedules could support production of a profile of patient groups potentially at risk of an ADE if prescribed that new drug in the context of their age, gender, comorbidities and/or co-medications. This risk information could then be communicated to prescribers, and would serve to promote caution until more is known about that new drug. This, in turn, would have the potential to reduce ADEs at the time when the risk is greatest.

Prospective identification of risk would make a valuable contribution to the health of the increasing numbers of older patients with multiple morbidities who are being co-prescribed new drugs soon after they are released.

## Competing interests

The authors declare that they have no competing interests.

## Authors' contributions

MW conceived the idea, worked with CP to design the study, and wrote the draft paper. SR designed and undertook all the statistical analyses of the hospital and pharmaceutical data. CP worked on the design of the study, provided interpretative feedback to MW and SR during the statistical analyses, and revised the draft paper. LE worked with MW and CP at the design stage, and revised the draft paper. All authors have read and approved the final manuscript.

## Authors' Information

MTW (PhD, M.Info.Mgt. & Systems Research) undertook work for this project when she was a Research Fellow at Deakin University. Her principal academic focus is on developing systems to support reduction of medication-related adverse events in older persons. CMP (PhD MFM MBBS FRACGP FACRRM FAICD FACHI) is Adjunct Associate Professor in General Practice, Monash University, and a practicing GP and Emergency Medicine specialist. He is Director of Research, Melbourne East General Practice Network, Visiting Fellow, Australian National University, and Clinical Lead, National E-Health Transition Authority. He joined the project as he was interested in how IT could be used to support reduction of medication-related adverse events in older patients in the primary care setting. SCR (BSc Hons) joined the project when he was Research Officer, School of Population Health, University of Western Australia. He has been involved in statistical analyses of linked administrative health data for over 10 years. EJE (PhD, M.A.) is Associate Professor, Sociology of Health, Deakin University. She has had a long-term involvement in improvements in public health and quality of life, and she was interested to see if we could develop a tool that would assist in improving the quality of life for older persons.

## Pre-publication history

The pre-publication history for this paper can be accessed here:

http://www.biomedcentral.com/1471-2458/11/361/prepub
